# High efficiency coronary MRA with non-rigid cardiac motion correction: beyond the quiescent period

**DOI:** 10.1186/1532-429X-18-S1-O107

**Published:** 2016-01-27

**Authors:** Jianing Pang, Yuhua Chen, Debiao Li

**Affiliations:** 1 Biomedical Imaging Research Institute, Cedars-Sinai Medical Center, Los Angeles, CA USA; 2 Bioengineering, University of California, Los Angeles, CA USA; 3 Computer and Information Science, University of Pennsylvania, Philadelphia, PA USA

## Background

Coronary arteries remain challenging structures to image using MRI due to the small size, tortuous course, and continual motion. Current high-resolution whole-heart techniques use prospective ECG-triggering and navigator gating to suppress cardiac and respiratory motion artifacts, respectively. However, these motion suppression strategies only accept data acquired within a narrow window in the cardiac and respiratory cycles, resulting in low scanning efficiency and prolonged scan time. Recent works use respiratory motion correction to achieve 100% respiratory gating efficiency [1–3]. In this work, we extend this concept and develop a non-rigid cardiac motion correction method to extend the cardiac acceptance window beyond the quiescent period.

## Methods

MR data was collected using a contrast-enhanced spoiled gradient echo sequence with 3D radial trajectory and retrospective cardiac and respiratory self-gating, from which 16 cardiac phases were reconstructed with affine respiratory motion correction [4]. Next, all diastolic phases are registered to mid-diastole using a symmetric diffeomorphic model [5]. Then, motion-corrected reconstruction is accomplished by inverting the encoding operator that includes sensitivity encoding and warping the target image to different cardiac phases [6]. Healthy subjects (N = 7) were scanned using a clinical 3T scanner (Siemens Verio). Three images are reconstructed from each dataset: mid-diastole quiescent window, extended window without motion correction, and extended window with motion correction. The scan efficiency, coronary sharpness, and apparent signal-to-noise ratio (aSNR) are compared using paired Student's t-test with a significance level of 0.05.

## Results

**Figure 1:**
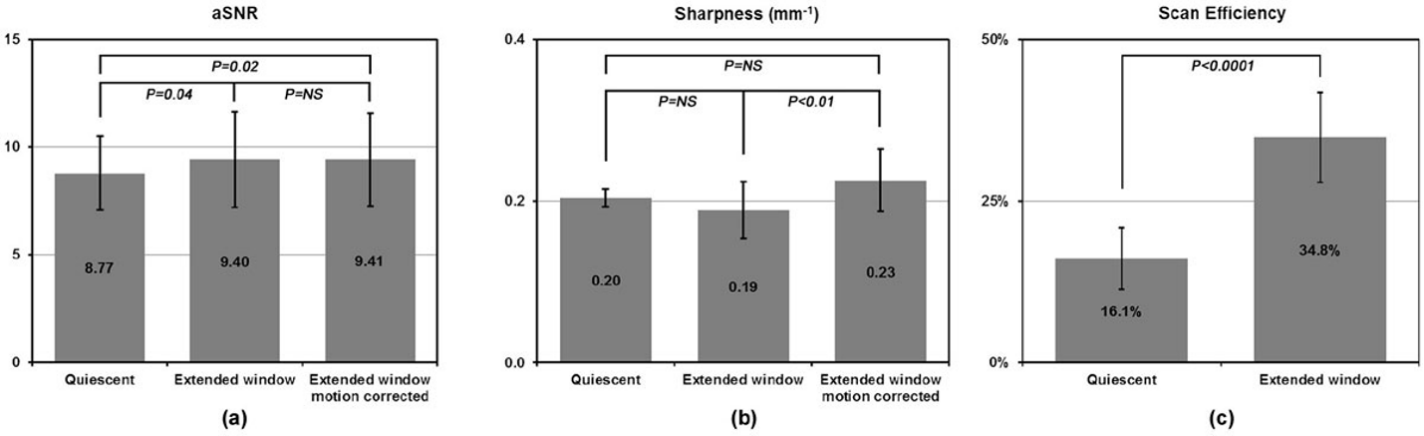
**Quantitative comparisons: (a) accepting more data with an extended window improves aSNR significantly over the mid-diastole window; (b) the residual cardiac motion within the wide acceptance window decreases the coronary sharpness, which is recovered by performing cardiac motion correction; (c) the proposed method significantly increases the imaging efficiency**.

**Figure 2:**
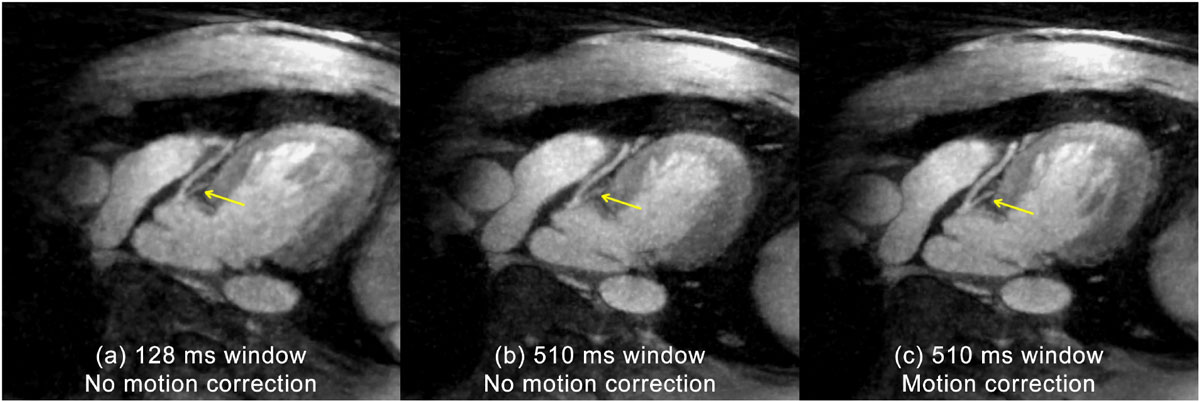
**An example dataset showing the effect of cardiac window size and motion correction: the mid-diastole window (a) minimizes residual motion but introduces blurring and decreases aSNR due to undersampling; an extended window (b) improves aSNR yet introduces motion blurring; the effect of cardiac motion is removed by the proposed cardiac motion correction technique (c)**.

Shown in Fig. 1, the proposed method offers high scanning efficiency, improves aSNR and maintains coronary sharpness over the mid-diastole reconstruction. Fig. 2 shows an example case: a typical mid-diastole window (128 ms) shows little motion blurring but considerable undersampling artifacts; a wide window covering the entire diastole (510 ms) increases aSNR but shows blurring due to cardiac motion; and the motion blurring is effectively removed with cardiac motion correction.

## Conclusions

In this work, we developed a cardiac motion correction framework for high-efficiency coronary MRA, allowing the cardiac acceptance window to be significantly widened while minimizing the artifacts from cardiac motion. The higher scan efficiency can be used to improve the image quality, as shown by our preliminary in vivo studies, or potentially reduce the scan time. Future developments will be focused on optimizing the registration parameters, and further validations on both healthy and patient subjects.
